# Quantification of risk factors for herpes zoster: population based case-control study

**DOI:** 10.1136/bmj.g2911

**Published:** 2014-05-13

**Authors:** Harriet J Forbes, Krishnan Bhaskaran, Sara L Thomas, Liam Smeeth, Tim Clayton, Sinéad M Langan

**Affiliations:** 1 Faculty of Epidemiology and Population Health, London School of Hygiene and Tropical Medicine, London WC1E 7HT, UK

## Abstract

**Objectives** To quantify the effects of possible risk factors for herpes zoster at different ages.

**Design** Case-control study.

**Setting** UK Clinical Practice Research Datalink primary care data.

**Participants** 144 959 adults diagnosed with zoster between 2000 and 2011; 549 336 age, sex, and practice matched controls.

**Main outcome measures** Conditional logistic regression was used to generate adjusted odds ratios to estimate the strength of association of each potential risk factor with zoster and assess effect modification by age.

**Results** The median age of the cases and controls was 62 years. Factors associated with increased risk of zoster included rheumatoid arthritis (3111 (2.1%) *v* 8029 (1.5%); adjusted odds ratio 1.46, 99% confidence interval 1.38 to 1.55), inflammatory bowel disease (1851 (1.3%) *v* 5118 (0.9%); 1.36, 1.26 to 1.46), chronic obstructive pulmonary disease (6815 (4.7%) *v* 20 201 (3.7%); 1.32, 1.27 to 1.37), asthma (10 243 (7.1%) *v* 31 865 (5.8%); 1.21, 1.17 to 1.25), chronic kidney disease (8724 (6.0%) *v* 29 437 (5.4%); 1.14, 1.09 to 1.18), and depression (6830 (4.7%) *v* 22 052 (4.0%); 1.15, 1.10 to 1.20). Type 1, but not type 2, diabetes showed some association with zoster (adjusted odds ratio 1.27, 1.07 to 1.50). The relative effects of many assessed risk factors were larger in younger patients. Patients with severely immunosuppressive conditions were at greatest risk of zoster—for example, patients with lymphoma (adjusted odds ratio 3.90, 3.21 to 4.74) and myeloma (2.16, 1.84 to 2.53), who are not eligible for zoster vaccination.

**Conclusions** A range of conditions were associated with increased risk of zoster. In general, the increased risk was proportionally greater in younger age groups. Current vaccines are contraindicated in people at the greatest risk of zoster, highlighting the need for alternative risk reduction strategies in these groups.

## Introduction

Herpes zoster (commonly known as shingles) is caused by the reactivation of latent varicella zoster virus when specific cell mediated immunity becomes compromised. It is a common disease in older people,^1^ with a lifetime risk of up to 30% rising to 50% among those living to 85 years.^2^ Zoster typically presents as a painful unilateral vesicular dermatomal rash that causes acute morbidity lasting two to four weeks.^3^ A severe complication—postherpetic neuralgia—although uncommon in patients aged under 50, develops in 12% of zoster patients aged 50 years or over^1^ ^4^; it causes intense pain that can last from months to years and is associated with considerable impairment of quality of life.

A live vaccine that reduces the risk of zoster and postherpetic neuralgia has recently been developed.^4^ The vaccine is licensed in peoople aged over 50 years. In the United States and Australia, it is recommended in people aged 60 years or over. In the United Kingdom, it is routinely available for patients aged 70, with a catch-up campaign for 72-79 year olds (on the basis of cost effectiveness studies^5^); the current catch-up cohort is those aged 79. Table 1 shows some examples of recommedations on vaccination in major countries. As the zoster vaccine is expensive, targeting vaccination towards groups at high risk of zoster is necessary. Age is the most important risk factor for zoster and postherpetic neuralgia, so it drives vaccination policies. The incidence of zoster rises from 3.5 per 1000 person years among 50-54 year olds to 7.1 per 1000 person years among 75-79 year olds in the UK.^6^ However, whether people with other risk factors, particuarly in younger age groups, might also benefit from vaccination is not clear.

**Table 1 tbl1:** Vaccine recommendations in United Kingdom, United States, Canada, Australia, and Sweden

Country	Groups recommended to receive vaccine	Contraindications
United Kingdom	People aged 70 years, with catch-up campaign for 72-79 year olds (current catch-up cohort is 79 year olds)	People with primary or acquired immunodeficiency state; active untreated tuberculosis infection; confirmed anaphylactic reaction to previous dose of varicella vaccine or any component of vaccine; pregnant women; people receiving immunosuppressive treatment (including high dose corticosteroids)
United States	Recommended for people aged ≥60 years	People with history of anaphylactic/anaphylactoid reaction to any component of vaccine; history of primary or acquired immunodeficiency state; people receiving immunosuppressive treatment, including high dose corticosteroids; women who are or may be pregnant
Canada	Recommended for people aged ≥60 years; also available for those aged 50-59 years	People with anaphylactic hypersensitivity to any of vaccine’s componenets; congenital or acquired immune deficiency; active untreated tuberculosis; use of immune suppressive drugs; pregnant women; people with certain acute illnesses
Australia	Recommended for people aged ≥60 years; also available for those aged 50-59 years	People with anaphylactic hypersensitivity to any of vaccine’s componenets; those receiving high dose systemic immunosuppressive treatment (such as chemotherapy, radiation therapy, or oral corticosteroids); people with malignant conditions of reticuloendothelial system; any person with similar immunosuppression due to disease or treatment
Sweden	Recommended for people aged ≥50 years	People with weakened immune systems due to drug treatment or other health conditions; allergy to gelatin or neomycin; moderate or severe illness; women who are or may be pregnant

Several clinical conditions not listed as contraindications for the zoster vaccine have been associated in some studies with an increased risk of zoster; these include autoimmune conditions such as rheumatoid arthritis,^7^ systemic lupus erythematosus,^8^ and inflammatory bowel disease,^9^ and chronic conditions such as diabetes mellitus,^10^ ^11^ chronic obstructive pulmonary disease,^11^ ^12^ chronic kidney disease,^13^ ^14^ ^15^ asthma,^16^ and depression.^2^ ^17^ Large, adequately powered studies investigating the association of these conditions with zoster are lacking.

This study therefore aimed to quantify the effects of various proposed risk factors for zoster and explore whether their effects differ by age group, to help to identify any groups of patients at high risk who are not currently targeted for vaccination. We aimed to add to the existing literature by using a very large data source and a consistent methodological approach to help comparison between various risk factors.

## Methods

We did a matched case-control study to quantify the effects of a range of individual level factors on the risk of zoster in a general population.

### Data source

This study used data from the UK Clinical Practice Research Datalink, a primary care database of anonymised patients’ records containing complete prescribing and diagnostic information and feedback from hospital referrals. Clinical diagnoses are coded in the Clinical Practice Research Datalink with Read codes.^18^ It is one of the largest sources of continuous patients’ records in the world, containing data on approximately 7% of the UK population, and is broadly representative of patients’ and practices’ characteristics in the UK.^19^ Sixty per cent of patients in the Clinical Practice Research Datalink have data available in the Hospital Episode Statistics database, which is a linked computerised database of hospital attendances in England from 1997.

### Selection of cases

The base study population consisted of all patients aged 18 years or over, under follow-up between 1 January 2000 and 31 December 2011, with no evidence of previous zoster (no codes for zoster or postherpetic neuralgia before the start of follow-up). We identified zoster cases by using the Clinical Practice Research Datalink and Hospital Episodes Statistics. Cases in the Clinical Practice Research Datalink were those with a zoster Read code and at least 12 months’ follow-up before a first diagnosis of zoster, to exclude past cases of zoster recorded retrospectively after registration at a general practice.^20^ Incident zoster in Hospital Episodes Statistics was identified by ICD-10 (international classification of diseases, 10th revision) codes (B02, B02.0, B02.1, B02.31, B02.7, B02.8, B02.9, G53.0) that appeared in the primary diagnosis field; we excluded patients with these codes recorded in secondary diagnosis fields, as this could reflect either incident zoster or sequelae of past zoster. For cases identified in Hospital Episodes Statistics, we took the date of hospital admission as the index date. If zoster was identified in both Hospital Episodes Statistics and the Clinical Practice Research Datalink, we took the earliest recorded zoster diagnosis as the index date.

### Selection of controls

We identified all potential controls for each case and used incidence density sampling to select up to four controls per case at random.^21^ Controls were registered with the practice at the index date of the case and for at least 12 months before and were matched to cases by practice, sex, and age (within 1 year). We selected controls before their exposure status for other risk factors was known. By matching on practice, we controlled for practice level socioeconomic status. Controls took the index date of their matched case. Controls had no history of zoster or postherpetic neuralgia at the index date, but they could go on to have zoster and therefore also be included as a case.^22^ Controls with no contact with their practice (that is, no consultation record of any kind, including repeat prescriptions and face to face or telephone consultations) any time from six months before to 12 months after the index date were assumed inactive in the database and excluded.

### Risk factors

Information on risk factors was based on records entered in the Clinical Practice Research Datalink any time before the index date. For each risk factor, we developed a definition and a list of relevant Read codes. A clinician reviewed all code lists, and these are available on request. Patients without Read codes for a clinical condition were assumed not to have the condition.

#### Key risk factors of interest

Our key risk factors of interest were rheumatoid arthritis, systemic lupus erythematosus, inflammatory bowel disease, chronic obstructive pulmonary disease, asthma, chronic kidney disease, depression, and diabetes (overall and by type) (see appendix section A for a full descriptions of how these risk factors were identified).

#### Severe immunosuppression

Our models included severely immunosuppressive conditions determined by the Advisory Committee on Immunization Practices to be contraindications to vaccination—namely, recent history (less than two years before index date) of leukaemia, lymphoma, or bone marrow transplant, or any history of HIV, haematopoietic stem cell transplantation, myeloma, or “other unspecified cellular immune deficiencies” (for example, pancytopenia).^23^ We also included use of immunosuppressive treatment; we extracted all relevant prescriptions before the index date and calculated duration of prescription (using data on quantity of tablets prescribed and numeric daily dose). We defined oral corticosteroid exposure as a 14 day course of high dose (≥20 mg/day) oral corticosteroids in the month before the index date. We included exposure to other immunosuppressive treatments in the month before the index date as an additional covariate (see appendix section A for further details).

#### Other characteristics

Other characteristics of patients included smoking (ex-smoker, current smoker, non-smoker), alcohol consumption (non-drinker, current drinker, ex-drinker), and body mass index (underweight (<18.5), normal weight (18.5-24.9), overweight (≥25), obese (≥30)) (see appendix section A for further details). We captured exposure to inhaled corticosteroids within three months before the index date (see appendix section A for further details), as some evidence suggests that they may increase the risk of zoster.^24^ We compared follow-up time (in years) for cases and controls, to check that the opportunity to record the exposures of interest in the two groups was equal.^25^

To explore whether the association between our risk factors of interest and zoster could be affected by ascertainment bias (that is, visiting a general practice for the risk factor may increase the likelihood of receiving a diagnosis of zoster), we investigated the association between zoster and treated epilepsy (an epilepsy Read code before index date and an epilepsy treatment (*British National Formulary* chapter 4.8.1) 12 months before the index date). Epilepsy is a chronic condition requiring high level healthcare use^26^; it is not thought to be associated with zoster.

### Statistical analysis

We described the characteristics of the study population by case-control status. The analysis accounted for the matched design by using conditional logistic regression, so all odds ratios accounted for the matching factors of age (within 1 year), sex, practice, and calendar time. Initially, we calculated univariable odds ratios to estimate the strength of association of each potential risk factor with zoster; 99% confidence intervals were generated to account for multiple testing and reduce the chance of picking up small and clinically unimportant associations. We then did multivariable analyses on patients with complete data for all variables (see also below for approach to missing data).

We investigated a potentially effect modifying role of age at diagnosis (index) date by calculating stratum specific odds ratios for each variable in the multivariable model by age groups (>50 years, 50-59 years, 60-69 years, and ≥70 years). We added interaction terms between age and other risk factors one at a time into the multivariable regression model and used likelihood ratio tests to investigate interactions.

To quantify the effect of our assessed risk factors, we estimated the age specific absolute rate of zoster for each factor by multiplying the age specific effect estimates by the age specific rate of zoster in the general population in 2010. We estimated the age specific general population rates within the Clinical Practice Research Datalink by dividing the number of incident cases of zoster in 2010 by the total person time contribution of the population at risk in that year, which included all patients in the Clinical Practice Research Datalink with at least one year of follow-up and no history of zoster.

We then evaluated to what extent combinations of risk factors could lead to a high risk of zoster, specifically within age groups not targeted for vaccination. We calculated the predicted risk of zoster for each person by summing the coefficients for their risk factors from the final model and multiplying their predicted odds ratio by the age specific rate of zoster in the general population. We were thus able to determine the number of cases aged under 70 but with a predicted risk of zoster equal to or higher than that of the general population aged at least 70 years (vaccination is currently offered to 70 and 79 year olds in the UK). We repeated this analysis with a 60 years age cut-off, to reflect vaccination policy from different countries. We restricted these calculations to people without severely immunosuppressive conditions.

Sensitivity analyses included use of a wider definition of exposure to oral corticosteroids and other immunosuppressive treatment (anyone exposed three months before the index date). We used multiple imputation by chained equations to account for missing data (11% of patients had missing data for alcohol or smoking).^27^ The imputation model included all covariates from the main outcome model, together with matching variables of age and sex, and extra comorbidities as additional markers of alcohol or smoking related diseases. We created five imputed datasets and combined them for analysis. We further adjusted for patient level socioeconomic status in the subgroup of patients registered at English practices for whom these data were available (see appendix sections B-D for further details). We applied two different definitions of an “active” control. Firstly, we considered controls to be inactive if they had no contact with their general practice during any time from one year before to two years after the index date. Secondly, we considered controls to be inactive if they had not consulted their general practice in the previous three years. Finally, we calculated the mean yearly consultation rate before the index date (by dividing the total number of face to face or telephone consultations during follow-up by the total years of follow-up before the index date) among patients with our risk factors of interest and among patients with epilepsy.

## Results

We identified a total of 145 397 incident cases of zoster. After matching, we removed inactive controls (n=27 928; 4.8% of all 577 264 matched controls). We excluded a small number of cases, as they had no eligible controls (n=438; 0.3%), largely owing to advanced age, leaving 144 959 cases.

Table 2 shows descriptive details of the cases and controls; 59.4% of cases and 61.0% of controls were female (the higher proportion among controls was due to the variable number of controls per case). The median age at diagnosis of zoster was 62 (interquartile range 48-73) years for cases and 62 (49-74) years for controls. Approximately 45% of zoster cases occurred in patients under 60 years and 65% in those under 70 years. Registered follow-up time before the index date was equivalent in cases and controls (8.6 (4.3-12.1) years).

**Table 2 tbl2:** Description of cases and controls. Values are numbers (percentages) unless stated otherwise

	Cases (n=144 959)	Controls (n=549 336)
**Matching factors**		
Sex:		
Female	86 071 (59.4)	335 272 (61.0)
Male	58 888 (40.6)	214 064 (39.0)
Age at index date (in years):		
18-29	10 849 (7.5)	38 761 ( 7.1)
30-49	28 762 (19.8)	104 708 (19.1)
50-59	27 833 (19.2)	105 157 (19.1)
60-69	31 134 (21.5)	121 108 (22.0)
70-79	28 025 (19.3)	110 097 (20.0)
80-89	15 891 (11.0)	61 566 (11.2)
≥90	2465 (1.7)	7939 (1.4)
Socioeconomic status (practice level)*:		
1 (least deprived)	28 938 (20.0)	109 663 (20.0)
2	28 853 (19.9)	109 253 (19.9)
3	29 811 (20.6)	112 888 (20.5)
4	30 550 (21.1)	115 678 (21.1)
5 (most deprived)	26 807 (18.5)	101 854 (18.5)
**Other characteristics**		
Mean (interquartile range) length of follow-up (in years)	8.6 (4.3-12.1)	8.6 (4.3-12.1)
Body mass index category:		
Underweight	2776 (1.9)	10 549 (1.9)
Normal weight	50 530 (34.9)	188 060 (34.2)
Overweight	47 886 (33.0)	177 603 (32.3)
Obese	29 581 (20.4)	109 440 (19.9)
Missing	14 186 (9.8)	63 684 (11.6)
Smoking status:		
Non-smoker	54 751 (37.8)	208 436 (37.9)
Current smoker	36 107 (24.9)	141 826 (25.8)
Ex-smoker	52 353 (36.1)	186 373 (33.9)
Missing	1784 (1.2)	12 701 (2.3)
Alcohol use:		
Non-drinker	14 481 (10.0)	56 774 (10.3)
Current drinker	103 113 (71.1)	383 976 (69.9)
Ex-drinker	12 786 ( 8.8)	45 242 (8.2)
Missing	14 579 (10.1)	63 344 (11.5)

*Measured by Index of Multiple Deprivation score.

Table 3 shows the proportion of cases and controls with our key risk factors of interest and other covariates. The strongest risk factors for zoster were the severely immunosuppressive conditions, although their overall prevalence was low—2.8% of cases and 1.2% of controls (table 3). Odds ratios varied from 1.78 to 13.46, although the latter estimate (for haematopoietic stem cell transplantation) had a particularly wide 99% confidence interval, as it was based on only 29 exposed patients.

**Table 3 tbl3:** Relative risk of zoster in patients with key risk factors of interest and other covariates

	No (%)			Odds ratio (99% CI)		
	Cases (n=144 959)	Controls (n=549 336)		Model 1*	Model 2†	Model 3‡
**Key risk factors of interest**						
Rheumatoid arthritis	3111 (2.1)	8029 (1.5)		1.52 (1.43 to 1.60)	1.46 (1.38 to 1.55)	1.22 (1.15 to 1.30)
Systemic lupus erythematosus	387 (0.3)	818 (0.1)		1.85 (1.58 to 2.17)	1.72 (1.45 to 2.04)	1.60 (1.35 to 1.90)
Inflammatory bowel disease	1851 (1.3)	5118 (0.9)		1.38 (1.29 to 1.48)	1.36 (1.26 to 1.46)	1.28 (1.18 to 1.38)
Chronic obstructive pulmonary disease	6815 (4.7)	20 201 (3.7)		1.34 (1.29 to 1.39)	1.32 (1.27 to 1.37)	1.22 (1.17 to 1.28)
Asthma	10 243 (7.1)	31 865 (5.8)		1.24 (1.20 to 1.28)	1.21 (1.17 to 1.25)	1.11 (1.06 to 1.16)
Chronic kidney disease	8724 (6.0)	29 437 (5.4)		1.20 (1.16 to 1.24)	1.14 (1.09 to 1.18)	1.12 (1.08 to 1.17)
Depression	6830 (4.7)	22 052 (4.0)		1.19 (1.15 to 1.24)	1.15 (1.10 to 1.20)	1.15 (1.10 to 1.19)
Diabetes	11 430 (7.9)	41 320 (7.5)		1.07 (1.04 to 1.10)	1.02 (0.99 to 1.05)	1.02 (0.99 to 1.05)
Diabetes type:§						
No diabetes	133 529 (92.1)	508 016 (92.5)		1.00 (1.00 to 1.00)	1.00	1.00
Type 1	396 (0.3)	1054 (0.2)		1.37 (1.18 to 1.60)	1.27 (1.07 to 1.50)	1.26 (1.06 to 1.49)
Type 2	10 359 (7.1)	38 136 (6.9)		1.05 (1.02 to 1.09)	1.01 (0.98 to 1.04)	1.01 (0.98 to 1.04)
Unknown	675 (0.5)	2130 (0.4)		1.20 (1.07 to 1.35)	1.13 (1.00 to 1.27)	1.12 (0.99 to 1.27)
**Other covariates**						
Inhaled corticosteroids	12 996 (9.0)	38 902 (7.1)		1.31 (1.28 to 1.35)	—	1.13 (1.08 to 1.18)
Severe immunosuppression:						
HIV	128 (0.09)	97 (0.02)		4.74 (3.34 to 6.73)	5.07 (3.41 to 7.54)	5.07 (3.41 to 7.54)
Leukaemia	205 (0.14)	368 (0.07)		2.14 (1.71 to 2.68)	1.78 (1.39 to 2.28)	1.77 (1.38 to 2.27)
Lymphoma	444 (0.31)	386 (0.07)		4.41 (3.68 to 5.28)	3.90 (3.21 to 4.74)	3.89 (3.20 to 4.73)
Myeloma	492 (0.34)	816 (0.15)		2.35 (2.03 to 2.73)	2.16 (1.84 to 2.53)	2.13 (1.82 to 2.51)
Haematopoietic stem cell transplantation	26 (0.02)	3 (0.00)		32.82 (6.80 to 158.44)	13.46 (2.68 to 67.60)	13.71 (2.73 to 68.94)
Other unspecified cellular immune deficiencies	95 (0.07)	190 (0.03)		1.90 (1.37 to 2.63)	1.57 (1.10 to 2.22)	1.49 (1.05 to 2.12)
Oral corticosteroids	2164 (1.49)	3822 (0.70)		1.82 (1.58 to 2.10)	—	1.48 (1.27 to 1.72)
Other immunosuppressive treatment	502 (0.35)	1058 (0.19)		2.20 (2.05 to 2.36)	—	1.82 (1.67 to 1.98)

*Adjusted for matching factors only.

†Adjusted for HIV, leukaemia, lymphoma, myeloma, haematopoietic stem cell transplantation, other unspecified cellular immune deficiencies, rheumatoid arthritis, systemic lupus erythematosus, inflammatory bowel disease, chronic obstructive pulmonary disease, asthma, chronic kidney disease, depression, diabetes, smoking, and alcohol.

‡Additionally adjusted for oral corticosteroids, other immunosuppressive treatment, and inhaled corticosteroids.

§Separate model run for diabetes type (no diabetes, type 1, type 2, unknown) instead of diabetes (yes/no).

Among our key risk factors of interest, systemic lupus erythematosus had the strongest relative risk of zoster (adjusted odds ratio 1.72, 99% confidence interval 1.45 to 2.04) (table 3), but the condition was very rare (0.3% of cases and 0.1% of controls). The risk of zoster was increased by more than 30% among patients with rheumatoid arthritis (adjusted odds ratio 1.46, 1.38 to 1.55) and chronic obstructive pulmonary disease (1.32, 1.27 to 1.37). Asthma, chronic kidney disease, and depression were associated with a greater than 10% increased risk of zoster. Although no association existed between zoster and diabetes overall (adjusted odds ratio 1.02, 0.99 to 1.05), we found strong evidence that patients classified as having type 1 diabetes were at increased risk of zoster (1.27, 1.07 to 1.50).

To assess whether some of the overall effect of these conditions was mediated by their treatments, we additionally adjusted for immunosuppressive treatments and inhaled corticosteroids (table 3, final column). The associations between zoster and rheumatoid arthritis, systemic lupus erythematosus, inflammatory bowel disease, chronic obstructive pulmonary disorder, and asthma were attenuated after adjustment but did not disappear. Among conditions not commonly treated with these drugs (chronic kidney disease, depression, and diabetes), the association did not vary after this additional adjustment.

Treated epilepsy, not expected to be associated with zoster, was marginally more common among cases than controls (1420 (0.98%) *v* 5142 (0.94%)). We found little evidence of association with zoster in crude (odds ratio 1.05, 0.97 to 1.14) or adjusted analyses (1.06, 0.97 to 1.15), suggesting that other associations observed in this study are unlikely to be explained by ascertainment bias.

We explored how the effect of our risk factors varied by age (tables 4 and 5). We found some evidence of age interaction. In general, the relative effects of these risk factors on zoster decreased with increasing age. For example, among patients with chronic kidney disease the odds ratio point estimate was 1.53, 1.21, and 1.11 among patients aged 18-49, 50-59, and 60-69 years respectively.

**Table 4 tbl4:** Numbers of cases and controls with various risk factors, stratified by age

Key risk factors of interest	<50 years			50-59 years			60-69 years			≥70 years	
	Case (n=39 611)	Control (n=143 469)		Case (n=27 833)	Control (n=105 157)		Case (n=31 134)	Control (n=121 108)		Case (n=46 381)	Control (n=179 602)
Rheumatoid arthritis	283	614		515	1282		867	2224		1446	3909
Systemic lupus erythematosus	122	146		99	181		73	207		93	284
Inflammatory bowel disease	473	1003		394	1080		424	1225		560	1810
Chronic obstructive pulmonary disease	99	304		614	1815		1850	5301		4252	12781
Asthma	3447	9931		1990	6303		2140	6851		2666	8780
Chronic kidney disease	403	862		613	1774		1544	5090		6164	21711
Depression	2692	7983		1474	4944		1079	3722		1585	5403
Diabetes	881	2383		1632	5283		3093	11253		5824	22401
Diabetes type:											
Type 1	259	576		81	240		38	142		18	96
Type 2	422	1246		1431	4727		2922	10633		5584	21530
Unknown	200	561		120	316		133	478		222	775

**Table 5 tbl5:** Association of various risk factors with herpes zoster, stratified by age

Key risk factors of interest	Adjusted odds ratio (99% CI)*				P value †
	<50 years	50-59 years	60-69 years	≥70 years	
Rheumatoid arthritis	1.69 (1.38 to 2.06)	1.45 (0.93 to 2.28)	1.49 (0.97 to 2.29)	1.41 (0.93 to 2.15)	0.203
Systemic lupus erythematosus	3.04 (2.14 to 4.31)	1.98 (0.86 to 4.58)	1.23 (0.52 to 2.89)	1.29 (0.56 to 2.93)	<0.001
Inflammatory bowel disease	1.73 (1.47 to 2.03)	1.40 (0.95 to 2.07)	1.30 (0.88 to 1.90)	1.18 (0.81 to 1.70)	<0.001
Chronic obstructive pulmonary disease	1.11 (0.80 to 1.54)	1.29 (0.65 to 2.53)	1.37 (0.71 to 2.66)	1.30 (0.68 to 2.51)	0.228
Asthma	1.24 (1.17 to 1.32)	1.19 (1.02 to 1.39)	1.22 (1.05 to 1.42)	1.18 (1.02 to 1.37)	0.465
Chronic kidney disease	1.63 (1.37 to 1.95)	1.26 (0.85 to 1.87)	1.14 (0.78 to 1.65)	1.10 (0.77 to 1.57)	<0.001
Depression	1.24 (1.16 to 1.33)	1.12 (0.94 to 1.33)	1.08 (0.90 to 1.30)	1.10 (0.93 to 1.30)	0.002
Diabetes	1.28 (1.15 to 1.43)	1.11 (0.87 to 1.42)	1.01 (0.80 to 1.28)	0.97 (0.77 to 1.22)	<0.001
Diabetes type:					
Type 1	1.51 (1.22 to 1.88)	1.16 (0.62 to 2.18)	0.98 (0.46 to 2.08)	0.62 (0.23 to 1.65)	<0.001
Type 2	1.22 (1.05 to 1.42)	1.09 (0.79 to 1.52)	1.02 (0.74 to 1.40)	0.97 (0.71 to 1.32)	
Unknown	1.20 (0.95 to 1.50)	1.30 (0.71 to 2.36)	1.02 (0.57 to 1.81)	1.07 (0.62 to 1.82)	

*Adjusted for HIV, leukaemia, lymphoma, myeloma, haematopoietic stem cell transplantation, other unspecified cellular immune deficiencies, rheumatoid arthritis, systemic lupus erythematosus, inflammatory bowel disease, chronic obstructive pulmonary disease, asthma, chronic kidney disease, depression, diabetes, smoking, and alcohol.

†P value for interaction.

To quantify the effect of our key risk factors of interest, particularly among age groups not targeted for vaccination, we estimated absolute rates of zoster (table 6). The rates remained low among patients aged 18-49 years (for example, for those with rheumatoid arthritis: 3.51 (99% confidence interval 2.40 to 5.13) per 1000 person years), despite the high relative risks in this age group. The absolute rates of zoster among patients aged 60-69 years were relatively high, particularly for those with chronic obstructive pulmonary disease and rheumatoid arthritis.

**Table 6 tbl6:** Estimated rate of zoster in patients with various risk factors, by age group

Key risk factors of interest	Rate of zoster/1000 person years (99% CI)			
	<50 years	50-59 years	60-69 years	≥70 years
General population (2010)	2.08 (1.74 to 2.49)	4.37 (3.72 to 5.12)	6.69 (5.76 to 7.76)	8.84 (7.49 to 10.43)
Rheumatoid arthritis	3.51 (2.40 to 5.13)	6.35 (3.46 to 11.66)	9.96 (5.57 to 17.77)	12.47 (6.94 to 22.41)
Systemic lupus erythematosus	6.32 (3.73 to 10.74)	8.67 (3.20 to 23.46)	8.20 (2.99 to 22.45)	11.36 (4.22 to 30.60)
Inflammatory bowel disease	3.59 (2.56 to 5.04)	6.13 (3.55 to 10.58)	8.67 (5.10 to 14.74)	10.41 (6.10 to 17.74)
Chronic obstructive pulmonary disease	2.31 (1.40 to 3.84)	5.62 (2.44 to 12.94)	9.19 (4.09 to 20.62)	11.54 (5.08 to 26.20)
Asthma	2.58 (2.03 to 3.28)	5.20 (3.81 to 7.11)	8.16 (6.04 to 11.00)	10.44 (7.64 to 14.25)
Chronic kidney disease	3.39 (2.38 to 4.85)	5.51 (3.17 to 9.59)	7.60 (4.52 to 12.78)	9.70 (5.74 to 16.37)
Depression	2.59 (2.03 to 3.31)	4.89 (3.51 to 6.80)	7.22 (5.19 to 10.05)	9.71 (6.94 to 13.58)
Diabetes	2.66 (1.99 to 3.56)	4.84 (3.23 to 7.27)	6.79 (4.62 to 9.97)	8.55 (5.76 to 12.70)
Diabetes type:				
Type 1	3.14 (2.14 to 4.67)	5.08 (2.32 to 11.16)	6.55 (2.66 to 16.12)	5.49 (1.75 to 17.21)
Type 2	2.54 (1.84 to 3.54)	4.77 (2.93 to 7.78)	6.79 (4.25 to 10.84)	8.54 (5.28 to 13.79)
Unknown	2.49 (1.68 to 3.74)	5.66 (2.66 to 12.07)	6.80 (3.28 to 14.07)	9.43 (4.67 to 19.03)

We calculated the predicted risk of zoster among 143 620 zoster cases (having excluded 1339 patients with severely immunosuppressive conditions) on the basis of their risk factor profile. Among these cases, 40 409 (28.1%) had at least one risk factor of interest. Of 97 789 zoster cases aged under 70 years, 3619 (3.7%) had a predicted risk as high as that for the general population aged 70 or over; 643 (0.96%) of 67 000 patients under 60 years had a predicted risk as high as those aged 60-69 years.

Sensitivity analyses imputing missing data for smoking and alcohol (see appendix section B), using different definitions for immunosuppressive treatment (see appendix section C), adjusting for patient level socioeconomic status (see appendix section D), and applying different definitions of an active control (see appendix section E) did not change the study findings. The consultation rate was similar among patients with epilepsy and those with our risk factors of interest (see appendix section F).

## Discussion

In this large matched case-control study, a range of conditions were associated with increased risk of zoster, including rheumatoid arthritis, systemic lupus erythematosus, inflammatory bowel disease, chronic obstructive pulmonary disease, asthma, chronic kidney disease, type 1 diabetes, and depression. The relative effects of many of these risk factors were larger among younger patients. This raises the question of whether vaccination of certain younger, high risk groups may be beneficial; cost effectiveness studies, also considering the risk of postherpetic neuralgia, would be needed to answer this question. Among a wide range of risk factors, people with contraindications to currently available vaccines were at the highest risk of zoster, highlighting the need to identify strategies to reduce the risk of zoster among these groups.

### Strengths and limitations of study

This large study included all incident cases of zoster in the Clinical Practice Research Datalink over an 11 year period, providing high power to detect small effects, except for rare potential risk factors. We assessed a wide range of factors, allowing adjustment for many of the known as well as potential confounders, in addition to exploring interactions with age, which is of key public health relevance in terms of vaccination policy.

The study may be subject to ascertainment bias; regular general practice visits for a chronic condition such as diabetes may increase the likelihood of receiving a zoster diagnosis. However, we believe that most zoster cases would present to their general practice, owing to the extensive rash and considerable pain associated with zoster and free general practice attendance in the UK.^28^ A survey about immunisation practices in the United States among people aged 60 and over found that 95% of those who knew they had zoster sought care.^29^ Furthermore, we found little evidence to suggest that patients with epilepsy (who were visiting their general practice as frequently as patients with our risk factors of interest) were at increased risk of zoster.

As with any observational study, residual confounding may be present. For example, exposure to varicella contacts, hypothesised to naturally boost varicella zoster virus specific immunity in people with latent varicella zoster virus infection and thus protect against zoster,^30^ was not available.

Some misclassification of zoster is possible. In UK primary care, diagnosis of zoster is clinically based with no laboratory testing. Validation of zoster diagnoses in primary care in the Netherlands found that 90.8% of cases had raised antibodies indicating recent zoster infection, suggesting that diagnosis of zoster has a high positive predictive value.^31^ The frequency with which general practitioners wrongly reject the diagnosis of zoster has not, however, been investigated; this may be more common among younger patients in whom zoster is uncommon. However, the most common misclassification of zoster is as recurrent herpes simplex, which very rarely presents in a dermatomal distribution.^32^ Zoster is typically a straightforward clinical diagnosis, so the extent of misclassification is likely to be minimal. Any misclassification is likely to be non-differential with respect to exposure status, thus leading to an underestimation of associations.

Finally, excluding “inactive” controls to remove patients who would not attend their general practice if they did develop zoster, may have resulted in healthier controls not being included and led to an underestimation of the odds ratios.

### Comparison with other studies

The strength of association between the autoimmune conditions in this study and risk of zoster is broadly in line with previous studies.^7^ ^8^ ^9^ ^33^ The increased risk associated with these conditions may partly reflect exposure to immunosuppressive drugs, as well as the conditions themselves. When we adjusted for immunosuppressive treatments, the increased risk of zoster was attenuated, suggesting that these drugs contribute to the overall increased risk of zoster in these patients.

The effect of chronic obstructive pulmonary disease is also consistent with previous studies.^11^ ^12^ After adjustment for oral and inhaled corticosteroids commonly used to treat chronic obstructive pulmonary disease, the association reduced in these patients, again suggesting that these treatments add to their overall risk of zoster. Larger associations between zoster and depression have been reported.^11^ ^17^ ^34^ No consensus exists on how to capture depression in electronic records; previous studies used different definitions, which may contribute to the varied findings. For example, a matched case-control study using commercial claims and encounters data in the United States found a higher risk of zoster among patients aged over 65 with depression (odds ratio 1.52, 95% confidence interval 1.46 to 1.58) but only included very severe cases (depressive disorder, chronic depressive personality disorder, or dysthymic disorder).^11^ The association of zoster with chronic kidney disease was also smaller than previous studies have suggested.^15^

The risks of zoster are reportedly increased in patients with diabetes of unspecified type.^10^ ^11^ ^34^ ^35^ We found some evidence that type 1, but not type 2, diabetes was associated with an increased risk of zoster (although differentiating between type was not based on diagnostic codes, so misclassification is possible). Patients with type 1 diabetes may be at higher risk of zoster owing to the immune dysregulation resulting from autoimmune pathogenesis.^36^ The lack of effect found with type 2 diabetes must be interpreted cautiously, as negative confounding by ethnic group is possible; the type 2 diabetes population may have included South Asian patients (known to be at lower risk of zoster^2^ ^37^ and higher risk of diabetes). Ethnicity is poorly recorded for older patients not recently registered in the Clinical Practice Research Datalink, so we could not capture this characteristic.

The observed increased risks of zoster suggest that all these conditions are associated with some cell mediated immunosuppression. Disease induced immunosuppression has been proposed in chronic obstructive pulmonary disease and asthma patients,^38^ ^39^ ^40^ ^41^ ^42^ although long term exposure to corticosteroids in these patients is also likely to contribute to immunosuppression. Patients with chronic kidney disease are known to have immune dysfunction,^43^ particularly when the disease reaches end stage. For depression, stress may increase the risk of zoster as it affects several neuroendocrine functions that can affect cell mediated immunity,^44^ and depression itself has been associated with poorer zoster immunity.^45^ ^46^

### Conclusions and policy implications

Severe immunosuppression is known to be associated with an increased risk of zoster.^11^ ^47^ ^48^ ^49^ What this study has highlighted, however, is that the strongest clinical risk factors for zoster are contraindications to its vaccine; the people arguably in most need of protection against zoster cannot currently benefit from vaccination. Alternative risk reduction strategies in these patients would help those at greatest risk of this disease and its complications.

Another important consideration regarding vaccination policy is whether the risk factors for zoster are also risk factors for zoster’s main morbidity, postherpetic neuralgia. To date, very few studies have looked at this research question, and most have been underpowered. The role of severe immunosuppression as a risk factor for postherpetic neuralgia is inconclusive; three studies found that “general” immunosuppression was a risk factor for postherpetic neuralgia,^35^ ^50^ ^51^ whereas another study found no association.^52^ Autoimmune conditions have scarcely been assessed; one large cohort study in 34 280 zoster patients identified in Taiwanese health insurance records found that those with systemic lupus erythematosus were at greater risk of postherpetic neuralgia (rate ratio 2.27, 95% confidence interval 1.75 to 2.94).^35^ Three studies found point estimates for the association between diabetes and postherpetic neuralgia greater than one in multivariate analyses, although the evidence was insufficient to confirm the association.^51^ ^53^ ^54^ Some evidence suggests that depression is associated with postherpetic neuralgia.^55^ Larger studies assessing these and other risk factors for postherpetic neuralgia are needed to improve our understanding of the risk factors for postherpetic neuralgia.

This study also raises the question of whether younger age groups at high risk of zoster may benefit from vaccination. Most cases of zoster occur in age groups too young for vaccination; this is because vaccination policy considers the risk of both zoster and postherpetic neuralgia, which is very uncommon in patients under 50. Deciding whether vaccination of groups of patients currently too young for vaccination would be cost effective will therefore require additional research on the risk factors for postherpetic neuralgia, specifically among patients aged under 50.

Our study has identified a range of conditions that are associated with an increased risk of zoster, raising the question of whether targeted zoster vaccination of specific high risk groups at younger ages is warranted. However, this study has also shown that patients at the greatest risk of zoster are not currently eligible to receive the vaccine.

**What is already known on this topic**

A new herpes zoster vaccination campaign introduced in the United Kingdom in September 2013 is focused on older people only
Recent literature has suggested that a range of clinical conditions are associated with an increased risk of zoster, raising the possibility that some younger people may be at high risk
However, large, highly powered studies investigating the association of these clinical conditions with zoster are lacking

**What this study adds**

Conditions associated with increased risk of zoster included rheumatoid arthritis, systemic lupus erythematosus, inflammatory bowel disease, chronic obstructive pulmonary disease, asthma, chronic kidney disease, type 1 diabetes, and depression
The increased risks were generally greater among younger age groups
The strongest risk factors are contraindications to vaccination, emphasising the need for alternative risk reduction strategies among these groups

## Notes

Ethical approval: Approval was obtained from the Clinical Practice Research Datalink Independent Scientific Advisory Committee (11_028A2RA) and the LSHTM Research Ethics Committee (A241 5930).

Transparency declaration: The lead author (the manuscript’s guarantor) affirms that this manuscript is an honest, accurate, and transparent account of the study being reported; that no important aspects of the study have been omitted; and that any discrepancies from the study as planned (and, if relevant, registered) have been explained.

Data sharing: No additional data available.

## Notes

Cite this as: *BMJ* 2014;348:g2911
