# Supplementary material for: Quantification of risk factors for herpes zoster: population based case-control study
Source: BMJ. 2014 May 13;348:g2911. doi: 10.1136/bmj.g2911 (PMC4019782; doi:10.1136/bmj.g2911)
Supplement: Supplementary file 1 [file forh018188.ww1_default.pdf]

Appendix

A: Further detail on defining risk factors
B: Dealing with missing data
C: Different definition of exposure to oral corticosteroids and immunosuppressive conditions
D: Association of various risk factors with zoster, after additionally adjusting for patient-level socioeconomic status
E: Different definitions of "active" controls
F: Consultation rate among patients with risk factors of interest

A: Further detail on defining risk factors

We assessed three autoimmune conditions; *rheumatoid arthritis, systemic lupus erythematosis and inflammatory bowel disease.* These were defined as a diagnosis prior to the index date. Chronic obstructive pulmonary disorder patients were defined as those with a diagnosis of chronic obstructive pulmonary disorder, including chronic bronchitis and emphysema, prior to the index date and ≥35 years at first chronic obstructive pulmonary disorder diagnosis). Asthma patients were those with an asthma diagnosis before the index date and an asthma-related prescription [short and long-acting beta-2 agonists and antimuscarinics, inhaled corticosteroids, cromoglycates and nedocromil, theophyllines, leukotriene receptor agonists and omalizumab] within 12 months prior to the index date; patients with a chronic obstructive pulmonary disorder diagnosis ever in their medical history were not classified as asthmatic). Chronic kidney disease patients were those with a diagnosis of mild, moderate or severe chronic kidney disease, kidney transplant or kidney dialysis, anytime prior to the index date. Depression was defined as having a diagnosis or symptom of depression (such as "feeling depressed" or "sad mood") or within one year prior to the index date; symptoms were included due to the trend post-2004 of using symptom rather than diagnosis codes in UK primary care[22].

To define diabetes we required a definite diabetes diagnosis, or a possible diabetes code [e.g. self-monitoring of blood glucose] with a subsequent diabetes-specific prescription [insulin or oral anti-diabetics], or ≥2 diabetes drug prescriptions prior to the index date; gestational diabetes and drug-induced diabetes were excluded. We also used age at first diagnosis, age at first treatment and treatment received to classify patients into Type 1 or 2 diabetes. Patients were categorised as type 1 or type 2 diabetes where possible. Distinguishing between type 1 and type 2 diabetes is not always possible from diabetes codes as patients are frequently given a non-specific code. Furthermore, where type of diabetes is assigned, it has been found to be unreliable.[1] Therefore we chose not to use this information, but instead use age at first diagnosis, age at first treatment and treatment received to classify diabetes type, as in previous Clinical Practice Research Datalink studies.[2] [3] Type 1 was assigned where; age at first diagnosis was ≤35 years and treatment ever was exclusively insulin, or patients received at least two insulin prescriptions ≤35 years, but had no diabetes diagnosis. Type 2 was assigned where; age at first diabetes diagnosis was >35; or patients received exclusively oral anti-diabetics's >35 years. Patients with age at diagnoses >35 but treated exclusively with insulin and those not fitting into these categories were assigned as "Unknown type".

Calculating duration of prescriptions in the Clinical Practice Research Datalink and identifying high-dose oral corticosteroids

In order to identify relevant prescriptions, it was necessary to calculate the duration of individual prescriptions prior to the index date.

Variables available

The Clinical Practice Research Datalink does not provide researchers with duration and dose for individual prescriptions. Instead, these must be generated using information from other variables. On prescribing, general practitioners select the drug and can enter information on quantity (of tablets or inhalers prescribed), number of days, number of packs, pack type (number of tablets per pack or number of puffs per inhaler) and also enter free text. The free text field contains the actual prescribing information; in other words how many tablets, grams, milligrams or puffs the patient should take each day. To utilize this prescribing information, the Clinical Practice Research Datalink developed an algorithm to derive a numerical value from the free text and provide this to researchers;[4] this is referred to within the Clinical Practice Research Datalink as the *numeric daily dose (NDD)*. The dose (in milligrams or micrograms) per tablet or puff is typically contained in the product name.

The completeness of each variable is described in table A1 below.

Table A1: Completeness of variables used to identify duration and dose of corticosteroid prescriptions before cleaning.

		**% complete**		
**Description**	**Variable name**	**Oral corticosteroids**	**Other immunosuppressive therapy**	**Inhaled corticosteroids**
Quantity[1]	Qty	99	99	99
Number of days	numdays	9	7	8
Number of packs	numpacks	2	3	1

Pack type[2]	packtype	<0.5	4	52
Dose in mg	*extract from product name*	100	100	100
Numeric daily dose	ndd	67	67	81

[1] For ICS prescriptions quantity referred to number of inhalers, otherwise quantity referred to number of tablets
[2] For ICS prescriptions pack type referred to numbers of puffs per inhaler, for OCS prescriptions pack type was largely missing

Data cleaning

We carried out a series of data-checking and data-cleaning tasks including; checking the accuracy of NDD for the 500 most commonly occurring free-texts; extracting data for pack type from other variables (eg quantity variable); checking the clinical and referral records of 20 randomly selected patients to check the duration and dose was consistent with the clinical picture; excluding implausible values of quantity and NDD.

As NDD was not always available it was necessary to impute missing data. A "hot-deck" style imputation method was adopted, which replaced missing data with comparable data from the same set. An algorithm was developed which reviewed each oral corticosteroid and other immunosuppressive therapy record and imputed missing values. An extra binary variable for quantity was created, categorising quantity about the median (42 tablets for steroids, 36 for other immunosuppressants) into low and high. If a patient had any other record with the same quantity and dose, the median NDD among those records was used where NDD was missing. If a patient had no recorded NDD but had any other record of the same dose and quantity as a binary variable, the median NDD among those records was used. If a patient did not have a recorded NDD or quantity, but had records for the same dose, then the median NDD among those records was used. If there was no record of NDD, dose or quantity, but there were other patients in the dataset in the same 5-year age band, of the same gender, with the same dose and quantity, the using median NDD for those records was used. Finally, if none of the above were possible, patients in the dataset in the

same 5-year age band, of the same gender, with the same dose and quantity as a binary variable, the median NDD among these records was used.

Pack type for inhaled corticosteroids was imputed using the most common pack type for the quantity and dose of each prescription. Where NDD was missing for inhaled corticosteroids, the median value of 4 puffs per day was used.

Calculating duration and dose

Using this information we calculated duration of oral corticosteroid or other immunosuppressive therapy prescription as follows: total quantity of tablets prescribed / NDD. Duration of inhaled corticosteroids was calculated: (quantity x pack type)/NDD. Dose was calculated as follow; NDD x dose per tablet or puff.

Algorithm to select smoking, alcohol and BMI status.

Data were derived from medical Read codes and data from the additional details file. Read codes classifying patients by BMI category are very rarely recorded, therefore were not used. Where patients had multiple recordings, the nearest status in the period -1y to +1month from index was taken (best); if not available, then the nearest in the period +1month to +1y after index was taken (second best); if not available, then the nearest before -1y from index was taken (third best); if not available, then take nearest after +1y from index was taken (least best).

B: Dealing with missing data

We used multiple imputation to account for missing data. Missing data was present for alcohol and smoking. In total, 89% percent of patients had complete data for all variables. To maximise the use of the data while properly incorporating the extra uncertainty arising due to missing data, multiple imputation by chained equations [5] was used to impute missing values for alcohol and smoking from multinomial models. The imputation model included all covariates from the main outcome model, together with the matching variables age and sex. We also included extra comorbidities, identified using medical Read codes, to look for additional markers of alcohol or smoking related diseases. These included: stroke, peripheral artery disease, angina (stable and unstable), acute coronary syndrome, congestive heart failure, myocardial infarction, hypertension and alcoholic liver disease (including portal hypertension) and pancreatitis. Five imputed datasets were created and combined for analysis. Distributions of imputed values were visually checked for comparability with the observed data.

Table B1: Relative risk of zoster in patients with various risk factors, using a multiply imputed dataset

	**Adjusted OR (99% CI)***
**Clinical conditions**	
Rheumatoid Arthritis	1.47 (1.39 to 1.56)
Systemic Lupus Erythematosis	1.71 (1.46 to 2.01)
Inflammatory Bowel Disease	1.35 (1.26 to 1.45)
COPD	1.34 (1.29 to 1.40)
Asthma	1.23 (1.20 to 1.27)
Chronic Kidney Disease	1.16 (1.12 to 1.20)
Depression	1.17 (1.13 to 1.22)
Diabetes	1.04 (1.01 to 1.07)
Diabetes Type	
No diabetes	1
Type 1	1.33 (1.14 to 1.56)
Type 2	1.02 (0.99 to 1.05)
Unknown	1.15 (1.03 to 1.29)

OR, odds ratio. CI, confidence interval. *Adjusted for HIV, Leukaemia, Lymphoma, Myeloma, HSCT, Other Immune deficiencies, RA, SLE, IBD, COPD, Asthma, CKD, Depression, Diabetes, Smoking and Alcohol.

C: Different definition of exposure to oral corticosteroids and immunosuppressive conditions

Rationale

The definition of exposure to oral corticosteroids and other immunosuppressive therapy in the main analysis was derived from guidelines on zoster vaccine contraindications (a 14-day course of high-dose oral corticosteroids or other immunosuppressive therapies, within the month prior to index date). The vaccine contraindications suggest patients remain immunosuppressed for one month following the end of their prescription. However we acknowledge this definition may not capture all patients with immunosuppression due to these medications.

Our sensitivity analysis therefore defined exposure as anyone taking an oral corticosteroid or other immunosuppressant within 3 months prior to the index date, and placed no restrictions on duration or dose of prescription.

Results

A much higher number of patients were defined as exposed to immunosuppressive therapy using this broader criterion (table C1). The overall effect of oral corticosteroids and immunosuppressive therapies was slightly lower when using the 3-month definition compared to the vaccine contraindication definition, however the confidence intervals overlapped (table C1). There were no major differences in the effect of our main risk factors after adjusting for the broader definition of exposure to immunosuppressive drugs, compared to the main analyses (table C2).

Table C1: Relative risk of zoster in patients taking immunosuppressive therapy, defined as exposure in the previous 3 months.

	Cases n (%)	Controls n (%)	Unadjusted OR (99% CI)	Adjusted OR (99% CI)
**Oral corticosteroid****	5304 (3.7)	12341 (2.3)	1.69 (1.62 to 1.77)	1.37 (1.31 to 1.44)
**Other immunosuppressant drugs ****	2361 (1.63)	4151 (0.76)	2.21 (2.07 to 2.36)	1.71 (1.57 to 1.86)

OR, odds ratio. CI, confidence interval. *Adjusted for HIV, Leukaemia, Lymphoma, Myeloma, HSCT, Other Immune deficiencies, Oral corticosteroids (previous 3 months), Other Immunosuppressive (previous 3 months), ICS, RA, SLE, IBD, COPD, Asthma, CKD, Depression, Diabetes, Smoking and Alcohol.
** Within 3 months prior to index date

Table C2: Relative risk of zoster in patients with various risk factors

	Adjusted OR (99% CI)*
**Clinical conditions**	
Rheumatoid Arthritis	1.17 (1.10 to 1.25)
Systemic Lupus Erythematosis	1.52 (1.27 to 1.82)
Inflammatory Bowel Disease	1.26 (1.17 to 1.37)
COPD	1.20 (1.14 to 1.26)
Asthma	1.11 (1.06 to 1.16)
Chronic Kidney Disease	1.11 (1.07 to 1.16)
Depression	1.15 (1.10 to 1.20)
Diabetes	1.01 (0.98 to 1.05)
Diabetes Type	
No diabetes	1
Type 1	1.25 (1.05 to 1.48)
Type 2	1.00 (0.97 to 1.03)
Unknown	1.11 (0.98 to 1.26)

OR, odds ratio. CI, confidence interval. *Adjusted for HIV, Leukaemia, Lymphoma, Myeloma, HSCT, Other Immune deficiencies, Oral corticosteroids (previous 3 months), Other Immunosuppressive (previous 3 months), ICS, RA, SLE, IBD, COPD, Asthma, CKD, Depression, Diabetes, Smoking and Alcohol.

D: Association of various risk factors with zoster, after additionally adjusting for patient-level socioeconomic status

Rationale

In the main analyses patients were matched on practice and thereby the analyses controlled for practice-level socioeconomic status. For patients registered at English practices and agreeing to their medical records being linked to other dataset, a patient level socioeconomic status score is available. Socioeconomic status (at the patient and practice level) is captured using quintiles of the Index of Multiple Deprivation score. At the patient level, the patient's home postcode is mapped at the lower level super output level to the corresponding 2007 IMD score; a low quintile represents the least deprived.

Results

In total, 427,689 (61.6%) patients had a patient-level socioeconomic status score. The results from our sensitivity analysis which additionally adjusts for patient-level socioeconomic status are shown in Table D1. There were no major differences compared to the main analyses.

Table D1: Relative risk of zoster in patients with various risk factors

	**Adjusted OR (99% CI)***
**Clinical conditions**	
Rheumatoid Arthritis	1.54 (1.42 to 1.65)
Systemic Lupus Erythematosis	1.82 (1.46 to 2.27)
Inflammatory Bowel Disease	1.39 (1.26 to 1.54)
COPD	1.33 (1.26 to 1.40)
Asthma	1.21 (1.16 to 1.26)
Chronic Kidney Disease	1.13 (1.08 to 1.19)
Depression	1.16 (1.10 to 1.22)
Diabetes	1.01 (0.98 to 1.06)
Diabetes Type	
No diabetes	1
Type 1	1.30 (1.05 to 1.61)
Type 2	1.01 (0.96 to 1.05)
Unknown	1.09 (0.93 to 1.27)

OR, odds ratio. CI, confidence interval. *Adjusted for HIV, Leukaemia, Lymphoma, Myeloma, HSCT, Other Immune deficiencies, RA, SLE, IBD, COPD, Asthma, CKD, Depression, Diabetes, Smoking and Alcohol and patient-level SES.

E: Different definitions of active controls

Rationale

In the main analyses we only included "active" controls, by ensuring controls had at least one consultation anytime within an 18 month period around the index date. In these sensitivity analyses, we applied different definitions of "active" controls. First we ensured controls had a consultation anytime from 1 year prior, to 2 years following the index date. Second, we required controls to have a consultation in the three years prior to index date.

Results

In the first analysis, 12,891 controls and 35 cases were excluded (compared to 27,928 controls and 88 cases in the main analysis). This meant 145,012 cases of zoster and 564,373 controls were included. In the second sensitivity analysis (where controls were required to have contact 3 years prior to index date), 31,551 controls and 303 cases were excluded; this left 144,744 cases of zoster and 545,713 controls in the analysis. There were no differences in the study findings compared to the main analyses (Table E1).

Table E1: Relative risk of zoster in patients with various risk factors

	Adjusted OR (99% CI)*once excluding controls without contact anytime from 1 year prior, to 2 years following the index date	Adjusted OR (99% CI)* once excluding controls without contact 3 years prior to index
**Clinical conditions**		
Rheumatoid Arthritis	1.47 (1.39 to 1.56)	1.45 (1.37 to 1.54)
Systemic Lupus Erythematosis	1.72 (1.46 to 2.04)	1.70 (1.43 to 2.01)
Inflammatory Bowel Disease	1.37 (1.28 to 1.48)	1.35 (1.26 to 1.46)
COPD	1.33 (1.27 to 1.38)	1.31 (1.26 to 1.36)
Asthma	1.23 (1.20 to 1.28)	1.21 (1.17 to 1.25)
Chronic Kidney Disease	1.14 (1.10 to 1.19)	1.13 (1.09 to 1.07)
Depression	1.17 (1.12 to 1.22)	1.15 (1.10 to 1.19)
Diabetes	1.03 (1.00 to 1.06)	1.02 (0.99 to 1.05)
Diabetes Type		
No diabetes	1	1
Type 1	1.32 (1.11 to 1.55)	1.29 (1.09 to 1.52)
Type 2	1.02 (0.98 to 1.05)	1.00 (0.97 to 1.04)
Unknown	1.15 (1.02 to 1.30)	1.13 (1.00 to 1.28)

OR, odds ratio. CI, confidence interval. *Adjusted for HIV, Leukaemia, Lymphoma, Myeloma, HSCT, Other Immune deficiencies, RA, SLE, IBD, COPD, Asthma, CKD, Depression, Diabetes, Smoking and Alcohol.

F: Consultation rate among patients with risk factors of interest

Rationale

We explored how frequently patients consulted the general practitioner as this may introduce ascertainment bias (i.e. patients visiting their general practitioner more frequently may be more likely to receive a zoster diagnosis). We calculated the mean yearly consultation rate prior to index date (by dividing the total number of face-to-face or telephone consultations during follow-up, by the total years of follow-up prior to index date) among patients with our risk factors of interest. We compared this to the mean consultation rate for epilepsy, to assess whether epilepsy patients had a similar likelihood of being diagnosed with zoster.

Results

The results are shown in Table F1. The consultation rates among patients with our risk factors of interest were very similar. The mean number of consultations per year among epilepsy patients was 10.2, suggesting these patients consult with similar frequency as patients with our risk factors of interest.

Table F1: Mean consultation rate prior to index date

	**Mean number of consultations per year prior to index date**
**Clinical conditions**	
Rheumatoid Arthritis	11.7
Systemic Lupus Erythematosis	10.9
Inflammatory Bowel Disease	9.7
COPD	11.7
Asthma	9.5
Chronic Kidney Disease	11.2
Depression	10.3
Diabetes	11.7

References

1. Royal College of General Practitioners, *Coding, Classification and Diagnosis of Diabetes*, NHS, Editor 2011.
2. Mulnier, H.E., et al., *Mortality in people with Type 2 diabetes in the UK.* Diabetic Medicine, 2006. **23**(5): p. 516-521.
3. Andersohn, F., et al., *Long-Term Use of Antidepressants for Depressive Disorders and the Risk of Diabetes Mellitus.* American Journal of Psychiatry, 2009. **166**(5): p. 591-598.
4. Shah, A.D. and C. Martinez, *An algorithm to derive a numerical daily dose from unstructured text dosage instructions.* Pharmacoepidemiol Drug Saf, 2006. **15**(3): p. 161-6.
5. White, I.R., P. Royston, and A.M. Wood, *Multiple imputation using chained equations: Issues and guidance for practice.* Statistics in medicine, 2011. **30**(4): p. 377-99.
